# Transcutaneous electrical acupoint stimulation reduces postoperative patients’ length of stay and hospitalization costs: a systematic review and meta-analysis

**DOI:** 10.1097/JS9.0000000000001598

**Published:** 2024-05-22

**Authors:** Yilong Liu, Jiefu Fan, Xiaoqing Zhang, Wenping Xu, Zhiwen Shi, Jiarong Cai, Peiqin Wang

**Affiliations:** a Department of Gastroenterology, Changzheng Hospital, Naval Medical University; b Naval Medical University; c Department of Emergency, Changhai Hospital, Naval Military Medical University, Shanghai, People’s Republic of China

**Keywords:** hospitalization costs, length of stay, meta-analysis, perioperative management, systematic review, transcutaneous electrical acupoint stimulation

## Abstract

**Objective:**

To study the effects of transcutaneous electrical acupoint stimulation (TEAS) on length of stay (LOS) and hospitalization costs in postoperative inpatients.

**Methods:**

Two researchers collectively searched PubMed, Embase, Cochrane Library, China Network Knowledge Infrastructure, and Wanfang Database. The search time was set from the beginning to 25 April 2023, to identify randomized controlled trials articles that met the criteria. Statistical analyses were performed using the Stata software (version 16.0). The risk of bias was assessed using the Cochrane risk-of-bias tool, and publication bias was evaluated using a funnel plot and Egger’s test. The quality of evidence was assessed according to the Grading of Recommendations Assessment, Development, and Evaluation approach.

**Results:**

Thirty-four randomized controlled trials were included. The main results showed that TEAS reduced hospitalization costs [standardized mean difference (SMD)=−1.92; 95% CI: −3.40, −0.43), LOS (SMD=−1.00; 95% CI: −1.30, −0.70) and postoperative LOS (SMD=−0.70; 95% CI: −0.91, −0.49] in postoperative patients. Subgroup analyses further revealed that TEAS was effective in reducing both the overall and postoperative LOS in patients undergoing multiple surgical procedures. It is worth noting that the observed heterogeneity in the results may be attributed to variations in surgical procedures, stimulation frequencies, and stimulation points utilized in different trials.

**Conclusions:**

TEAS can help postoperative patients reduce their LOS and hospitalization cost. However, considering the bias identified and heterogeneity, the results of this review should be interpreted with caution.

## Introduction

Highlights

- Transcutaneous electrical acupoint stimulation (TEAS) can reduce hospitalization costs.
- TEAS can reduce postoperative length of stay.
- TEAS can reduce length of stay.

Transcutaneous electrical acupoint stimulation (TEAS) is a noninvasive treatment method that involves the application of electrodes to specific acupoints on the patient’s skin^1^. The purpose of this technique is to deliver electrical stimulation at a predetermined intensity comparable to that of conventional electro-acupuncture^1^. TEAS has increasingly gained recognition in clinical settings and is now considered an essential component of perioperative management, primarily due to its lack of drug-related side effects and noninvasive characteristics^2^.

In recent years, there has been a surge in studies across various medical departments aimed at exploring the potential of TEAS to enhance the recovery of postoperative patients^3–6^. Research findings indicate that TEAS can facilitate postoperative recovery in various ways, including pain relief, regulation of gastrointestinal function, reduction of inflammation, management of stress, and prevention of postoperative cognitive dysfunction^7^. The length of stay (LOS) and hospitalization costs are crucial indicators for evaluating patients’ postoperative recovery^3,8,9^. Considering the increasing influence of LOS and hospitalization costs on the decision-making of both physicians and patients, especially in China where there is a significant conflict between rising healthcare demands and limited resources, it is essential to understand the economic value of perioperative TEAS for inpatients who have undergone surgery^10^. The impact of TEAS on LOS and hospitalization costs for postoperative inpatients is yet to be clearly elucidated^3,11–13^. To date, there has been a lack of systematic reviews or meta-analyses that specifically explore the influence of TEAS on postoperative hospitalization, with LOS and hospitalization costs as the primary outcome measures.

Thus, the objective of this review was to assess and analyze the effects of TEAS on LOS and hospitalization costs among postoperative inpatients, with the aim of offering guidance for clinical application and future research endeavors.

## Materials and methods

### Study design and protocol

The work has been reported in line with Preferred Reporting Items for Systematic Reviews and Meta-Analyses (PRISMAP) (SDC, Table 1, Supplemental Digital Content 1, http://links.lww.com/JS9/C606, Supplemental Digital Content 2, http://links.lww.com/JS9/C607) and Assessing the methodological quality of systematic reviews (AMSTAR, Supplemental Digital Content 3, http://links.lww.com/JS9/C608) Guidelines^14,15^. Prior to data extraction, we registered our review in the PROSPERO database.

### Search strategy

Two independent researchers conducted a thorough literature search to ensure the inclusion of as many relevant studies as possible. The following electronic databases were searched: PubMed, Embase, Cochrane Library, China Network Knowledge Infrastructure (CNKI), and Wanfang Database. The search terms included transcutaneous electrical acupoint stimulation, transcutaneous acupoint electrical stimulation, TEAS, hospital stay, length of stay, length of hospitalization, hospitalization time, hospitalization costs, and hospitalization expenses. The search covered all relevant studies from the inception of the databases to 25 April 2023. In addition, we reviewed the references of the selected studies to identify any potential additional relevant studies. For the retrieval strategy, a combination of medical subject heading (MeSH) terms and free words was used. The complete search strategy used for each database is shown in Table S2.

### Inclusion criteria

Only prospective randomized controlled trials (RCTs) and peer-reviewed articles were eligible for inclusion in this meta-analysis. The participants included in the studies had to be inpatients who had recently undergone surgery without any restrictions based on age, sex, or nationality. For an RCT to be considered, it had to contain both a study group and a control group and provide quantifiable outcome indicators such as LOS, postoperative LOS, and hospitalization costs. In cases where multiple studies reported the same topic, only the most recent publication was included in the meta-analysis.

### Exclusion criteria

The following types of articles were excluded from the analysis: reviews, case reports, and nonrandomized controlled trials (non-RCTs). Articles were excluded if they did not provide a description of TEAS or measurable outcome indicators. Studies that explored different forms of acupuncture, such as acupoint injection, suture embedding, transcutaneous electrical nerve stimulation, acupuncture combined with oral Chinese medicine, and other nonacupuncture-related therapies, were also excluded.

### Article screening and data extraction

The identified articles were screened by two reviewers, who evaluated them based on predetermined inclusion and exclusion criteria. Full-text versions of articles that met the criteria were obtained. Data from the selected articles were extracted and entered into a predefined spreadsheet. The extracted data included information such as author, publication year, sample size, type of surgery, acupoint selection, timing of the stimulus, frequency of the stimulus, and magnitude of the stimulus current, among other relevant details. In case of any discrepancies or disagreements between the reviewers, a third reviewer was consulted to resolve them.

### Quality assessment

Two reviewers independently utilized the Cochrane tool to evaluate articles for their risk of bias (RoB), and assessed the quality of the studies using the Grading of Recommendations Assessment, Development, and Evaluation (GRADE) methodology. In cases where there were discrepancies between the assessments of the two reviewers, a third reviewer was consulted to effectively address and resolve any disagreement.

### Statistical analysis

A random-effects meta-analysis was used to compute the standardized mean difference (SMD) in LOS, postoperative LOS, and hospitalization cost estimates. Subgroup analysis was carried out to analyze the different effects of TEAS on the common types of surgeries, including gastrointestinal surgery, lung surgery, orthopedic surgery, urological surgery, gynecological surgery, endoscopic surgery, and surgery for the elderly.

*I* ^2^ statistics and the Cochrane *Q* test were used to examine the heterogeneity among the effect estimates. The heterogeneity of the pooled estimates with *P*<0.10 (Cochrane *Q* test) was deemed significant. *I* ^2^ statistics of 0–25%, 25–50%, and >50% indicated low, moderate, and high heterogeneity, respectively. Funnel plots and Egger’s test were used to evaluate potential publication bias and the Trim and Fill method was used to examine the sensitivity of the results to publication bias. Sensitivity analyses were performed to separate studies by stimulus acupoints, stimulus frequency, and quality. We further examined the influence of individual estimates on pooled RRs using leave-one-out analysis. Statistical analyses were performed using the Stata software (version 16.0).

## Results

### Search and study selection results

**Figure 1:**
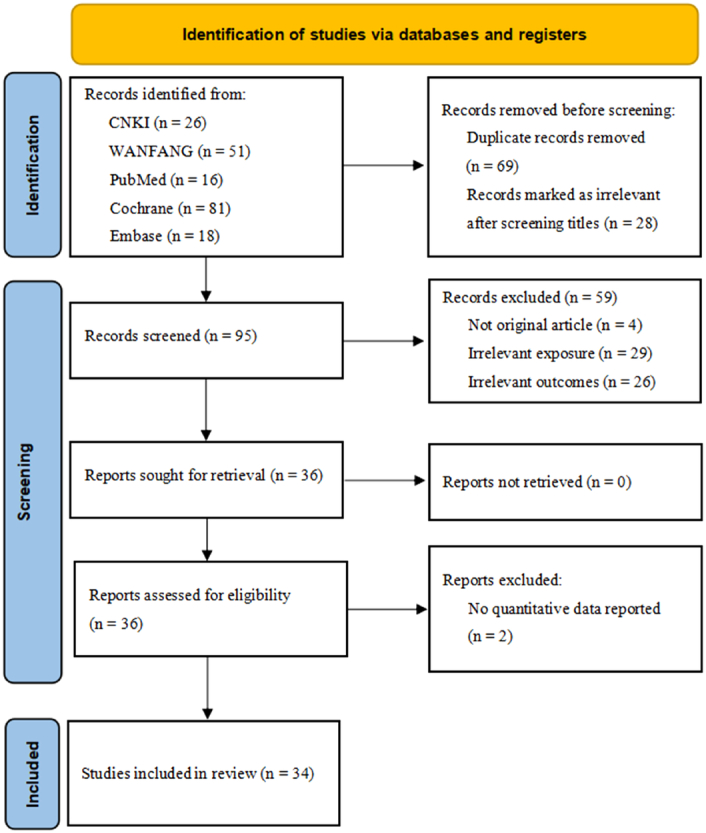
Flow chart of literature screening.

A total of 192 studies were identified using five databases. After removing replicates, 123 potentially relevant studies were identified. A total of 87 studies were excluded after reading the titles and abstracts. The full-text of the remaining 36 studies was assessed, and 2 studies were excluded according to the inclusion or exclusion criteria. Finally, 34 studies were included in the meta-analysis^5,6,9,11–13,16–43^. The detailed process is illustrated in Figure 1.

### Study characteristic

The included studies were published from 2015 to 2023 and included 26 (76.5%) articles from the last 5 years. A total of 2980 patients were included in the study, including 1500 in the TEAS group and 1480 in the control group. These studies included 14 gastrointestinal surgeries, 6 gynecological surgeries, 6 lung surgeries, 3 orthopedic surgeries, 2 urological surgeries, and 1 cardiovascular surgery. In addition, two studies involved multiple surgical systems. The specific characteristics of the included studies are presented in Table 1.

**Table 1:** Basic characteristics of the study articles.

		TEAS group			Control group							
Study ID	Type of surgery	Sample size	Age	Sex(Male/Female)	Sample size	Age	Sex(Male/Female)	Acupoint selections	Stimulus timing (Pr/In/Po)	Stimulus frequency	Stimulus current magnitude	GRADE
Chen *et al*., 2021^16^	Gastrointestinal surgery	36	45.36±5.21	21/15	36	47.13±5.24	20/16	Zusanli(ST36) + Neiguan(PC6)	Pr + In + Po	2/100 HZ	8–12 mA	Moderate
Deng *et al*., 2019^17^	Urological surgery	30	40.5±6.4	0/30	30	39.4±7.4	0/30	Zusanli (ST36) + Sanyinjiao (SP6) + Neiguan (PC6) + Hegu (LI4)	Pr + Po	2 HZ	^a^	Moderate
Du, 2015^18^	Lung surgery	46	56.2±12.6	21/25	44	55.9±13.5	23/21	Neiguan (PC6) + Hegu (LI4)	Pr + In + Po	2/100 HZ	15–25 mA	Moderate
Ge *et al*., 2021^19^	Orthopedic surgery	42	72.5±6.8	22/20	42	73.2±7.2	23/19	Neimadian (Extra) + Zusanli (ST36) + Yanglingquan (GB34) + Xuehai (SP10) + Yinlingquan (SP9)	Po	2/100 HZ	15–25 mA	Moderate
Li *et al*., 2020^20^	Gynecological surgery	60	53.4±11.6	0/60	60	52.8±13.7	0/60	Hegu (LI4) + Neiguan (PC6) + Sanyinjiao (SP6)	Po	2/100 HZ	^a^	Moderate
Lin *et al*., 2020^21^	Gynecological surgery	30	38.30±7.9	0/30	30	35.23±7.8	0/30	Neiguan (PC6) + Hegu (LI4) + Zusanli (ST36) + Sanyinjiao (SP6)	Pr + Po	2/100 HZ	^a^	Moderate
Zhao *et al*., 2020^13^	Gastrointestinal surgery	63	63.1 ± 8.1	48/15	49	62.3 ± 7.2	39/10	Feiyu (BL13) + Hegu (LI4) + Zusanli (ST36)	Pr	100 HZ	20–25 mA	Moderate
Liu *et al*., 2020^22^	Gastrointestinal surgery	30	51.2±5.0	18/12	30	51.0±5.2	20/10	Hegu (LI4) + Laogong (PC8) + Zusanli (ST36)	Pr + In + Po	2/100 HZ	8-10 mA	Moderate
Lv *et al*., 2022^23^	Multiple operations	52	37.17±10.59	0/52	51	34.63±9.03	0/51	Neiguan (PC6) + Zusanli (ST36)	Po	2/100 HZ	6–9 mA	Moderate
Sun *et al*., 2020^24^	Gynecological surgery	39	23.4±2.8	0/39	39	24.2±3.5	0/39	Hegu (LI4) + Neiguan (PC6)	Pr + In	2/15 HZ	10–30 mA	Moderate
Tang *et al*., 2020^25^	Gastrointestinal surgery	30	52.5±2.5	17/13	30	52.1±2.3	16/14	Zusanli (ST36) + Shangjuxu (ST37) + Xiajuxu (ST39) + Neiguan (PC6)	Pr + In	2/100 HZ	^a^	Moderate
Wang *et al*., 2021^27^	Lung surgery	40	67.6±7.6	24/16	40	67.0±7.2	26/14	Neiguan (PC6) + Hegu (LI4)	Pr + In	2/100 HZ	6–10 mA	Moderate
Wang *et al*., 2021^27^	Gynecological surgery	40	67.5±7.4	0/40	40	67.1±7.0	0/40	Neiguan (PC6) + Hegu (LI4)	Pr + In	2/100 HZ	6 mA	Moderate
Kong *et al*., 2019^28^	Lung surgery	25	56±11	Unknown	25	53±10	Unknown	Hegu (LI4) + Neiguan (PC6) + Houxi (SI3) + Zhigou (TE6)	Pr + In	2/100 HZ	6–12 mA	High
Yang *et al*., 2018^29^	Gastrointestinal surgery	45	73.34±12.14	18/27	45	71.22±13.46	25/20	Zusanli (ST36) + Shangjuxu (ST37)	Po	Unknown	Unknown	Moderate
Yuan *et al*., 2017^30^	Gastrointestinal surgery	30	54.9±9.8	12/18	30	54.6±10.4	16/14	Neiguan (PC6) + Hegu (LI4) + Zusanli (ST36)	Pr + In + Po	Unknown	^a^	High
Zhang *et al*., 2018^31^	Orthopedic surgery	30	68±7	26/4	30	65±5	25/5	Hegu (LI4) + Neiguan (PC6)	Pr + In	2/100 HZ	^a^	Moderate
Duan *et al*., 2020^32^	Lung surgery	30	66.5±4.3	23/7	30	67.0±3.8	22/8	Neiguan (PC6) + Zhigou (TE6) + Houxi (SI3)	Pr + In	2/100 HZ	10–15 mA	High
Wang *et al*., 2017^33^	Gastrointestinal surgery	30	69.1±4.5	17/13	30	68.5±2.1	18/12	Zusanli (ST36) + Hegu (LI4) + Feiyu (BL13)	Pr + In	2/100 HZ	8–12 mA	Moderate
Wei *et al*., 2019^34^	Gastrointestinal surgery	52	Unknown	Unknown	52	Unknown	Unknown	Hegu (LI4) + Quchi (LI11) + Neiting (ST44) + Zusanli (ST36)	Po	20 HZ	Unknown	High
Wu *et al*., 2022^35^	Orthopedic surgery	44	56±8	Unknown	40	56±8	Unknown	Zusanli (ST36) + Sanyinjiao (SP6)	Pr + In + Po	2/15 HZ	Unknown	Moderate
Yang *et al*., 2020^36^	Cardiovascular surgery	50	6.9±2.4	31/19	50	7.3±2.2	32/18	Baihui (GV20) + Neiguan (PC6) + Hegu (LI4) + Ximen (PC4)	In	2/100 HZ	6 mA	Moderate
Wang *et al*., 2020^37^	Gastrointestinal surgery	50	47±8	34/16	50	48±8	33/17	Neiguan (PC6) + Hegu (LI4)	Pr + In	2/100 HZ	6–10 mA	Moderate
Huang *et al*., 2019^38^	Lung surgery	20	47±15	11/9	20	45±14	10/10	Feiyu (BL13) + Hegu (LI4) + Neiguan (PC6)	Pr + In	2/100 HZ	5–15 mA	Moderate
Wang *et al*., 2020^39^	Gastrointestinal surgery	35	48±12	16/19	35	48±11	14/21	Hegu (LI4) + Neiguan (PC6)	Pr + In	2/100 HZ	^a^	Moderate
Li *et al*., 2023^40^	Multiple operations	79	57±10	53/26	79	56±9	60/19	Liangmen (ST21)+Daheng (SP15)	Po	2–5 HZ	^a^	High
Mu *et al*., 2019^41^	Gynecological surgery	55	24±3	0/55	55	24±3	0/55	Zusanli (ST36)	Po	30/60 HZ	15–20 mA	Moderate
Fan *et al*., 2018^42^	Gastrointestinal surgery	26	54±7	13/13	26	54±8	13/13	Neiguan (PC6) + Hegu (LI4) + Zusanli (ST36) + Shangjuxu (SP6) + Xiajuxu (ST39)	Pr + In	2/100 HZ	3–8 mA	Moderate
Lu *et al*., 2022^43^	Gastrointestinal surgery	47	56.2±9.0	26/21	47	55.6±9.9	23/24	Neiguan (PC6) + Zusanli (ST36)	Pr + Po	2/10 HZ	4–11 mA	High
Tu *et al*., 2018^5^	Lung surgery	72	64.34±8.25	42/30	72	62.88±8.37	39/33	Feiyu (BL13) + Hegu (LI4) + Zusanli (ST36)	Pr + In	2/100 HZ	5–30 mA	High
Zhou *et al*., 2018^6^	Gynecological surgery	43	32.2±3.5	0/43	45	30.6±3.9	0/45	Zusanli (ST36) + Sanyinjiao (SP6)	Pr + Po	2/10 HZ	7–11 mA	High
Huang *et al*., 2019^38^	Gastrointestinal surgery	29	58.59±11.27	12/17	28	60.57±12.1	17/11	Zusanli (ST36)	Pr + In	2/10 HZ	^a^	High
Li *et al*., 2021^11^	Gastrointestinal surgery	140	60±13	85/55	140	62±12	96/44	Hegu (LI4) + Neiguan (PC6) + Zusanli (ST36) + Shangjuxu (SP6)	Pr + In + Po	2/100 HZ	^a^	High
Que *et al*., 2021^12^	Urological surgery	30	45.4±18.7	18/12	30	47.1±16.3	19/11	Shenyu (BL23) + Yinlingquan (SP9) + Hegu (LI4) + Neiguan (PC6)	Pr	2/100 HZ	5–30 mA	High

^a^ The maximum intensity tolerated by the patient.

In, intraoperative; Po, postoperative; Pr, preoperative.

### Synthesis of the results

**Figure 2:**
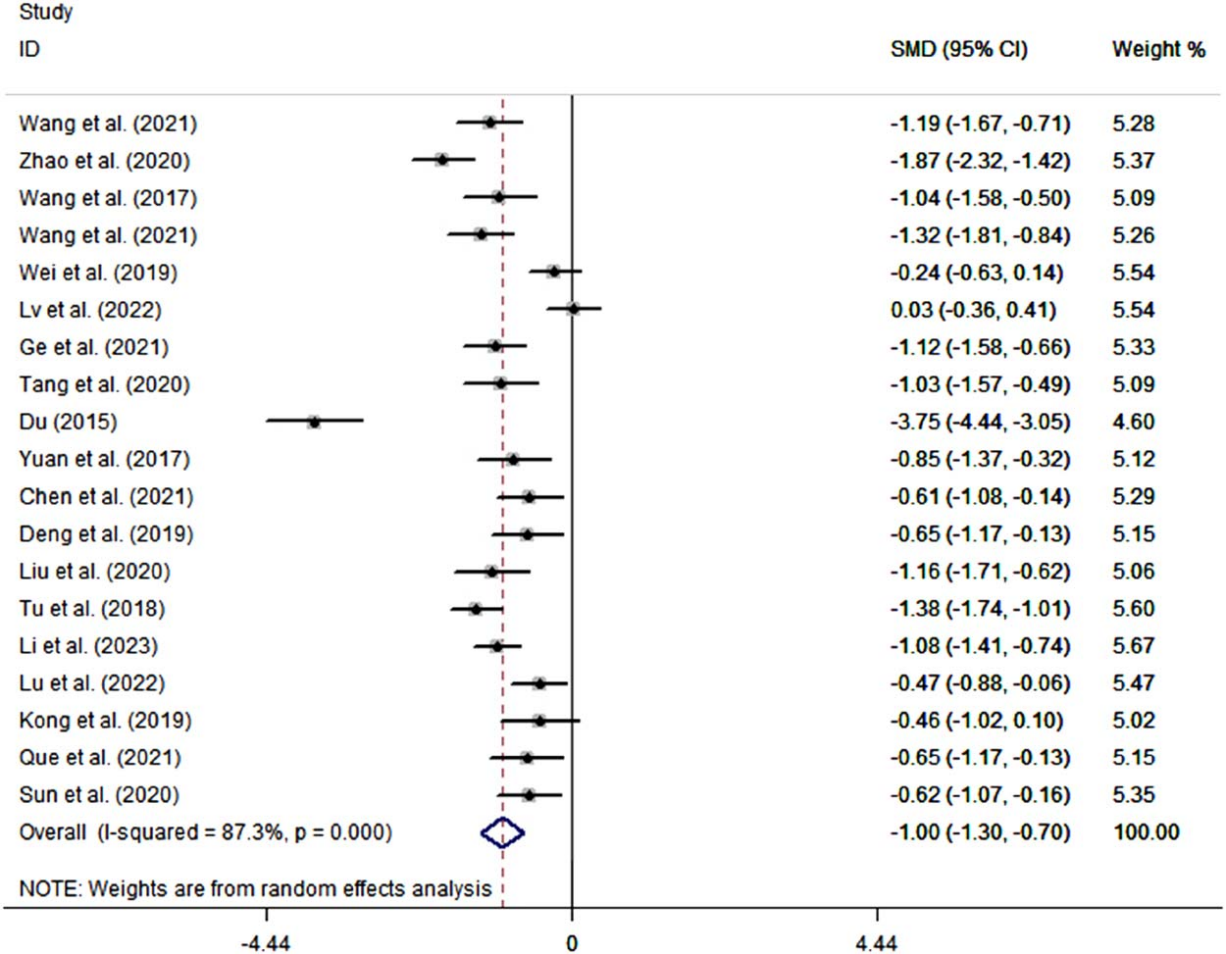
Forest plot of the length of stay.

**Figure 3:**
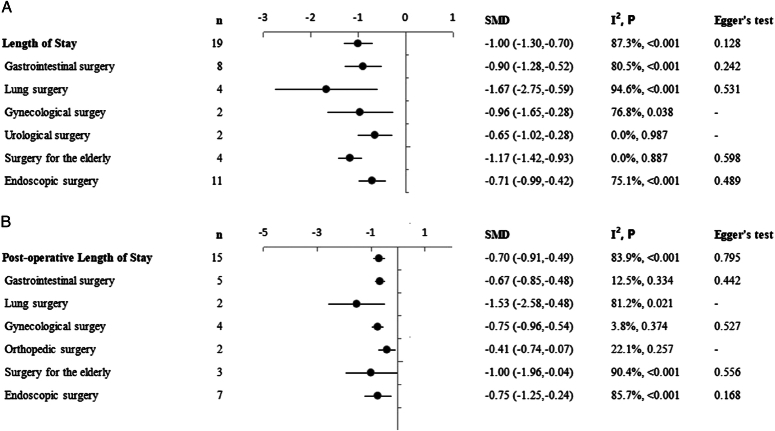
Effect of TEAS on length of stay and post-operative length of stay. Forest plot of subgroup analysis the effect of TEAS on length of stay (A) and post-operative length of stay (B).

**Figure 4:**
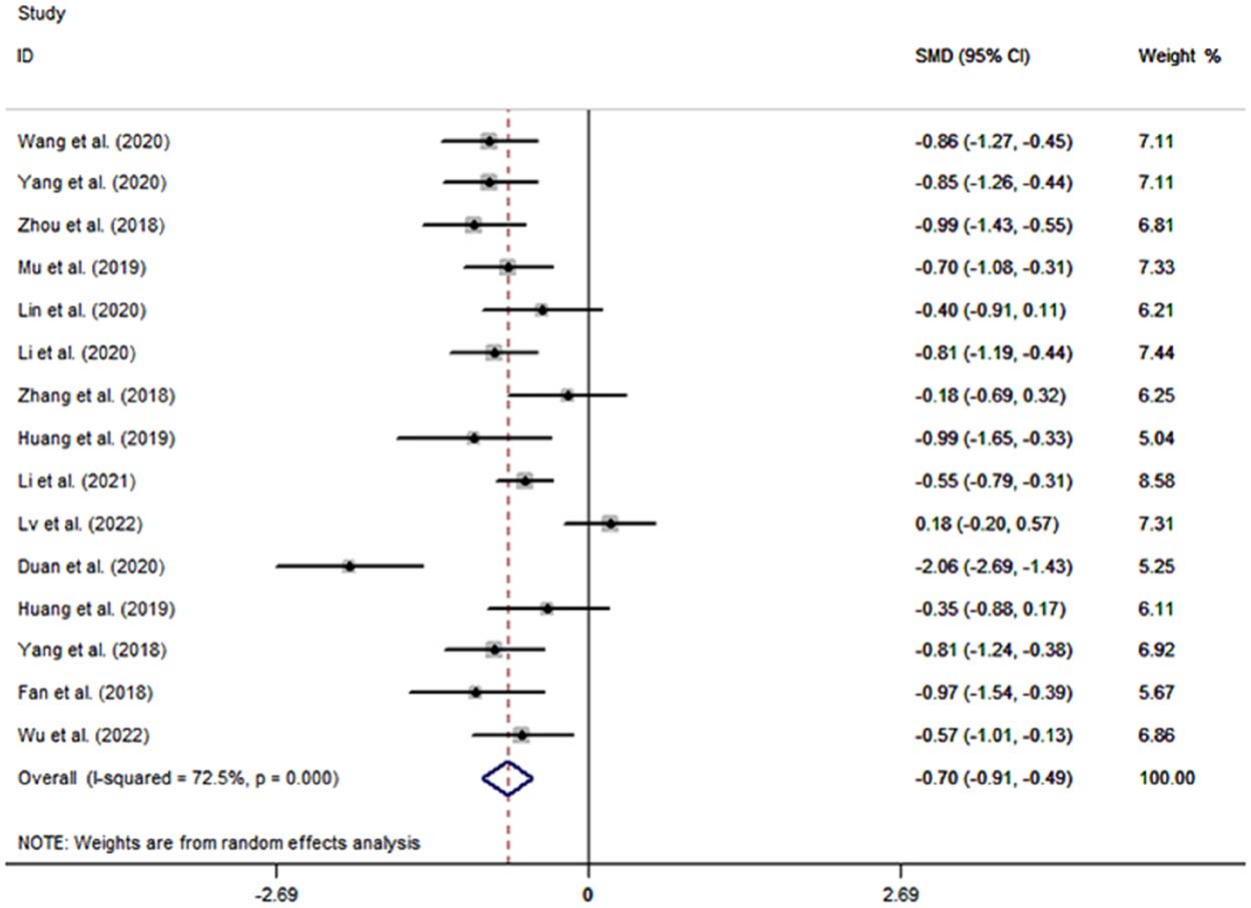
Forest plot of the postoperative length of stay.

**Figure 5:**
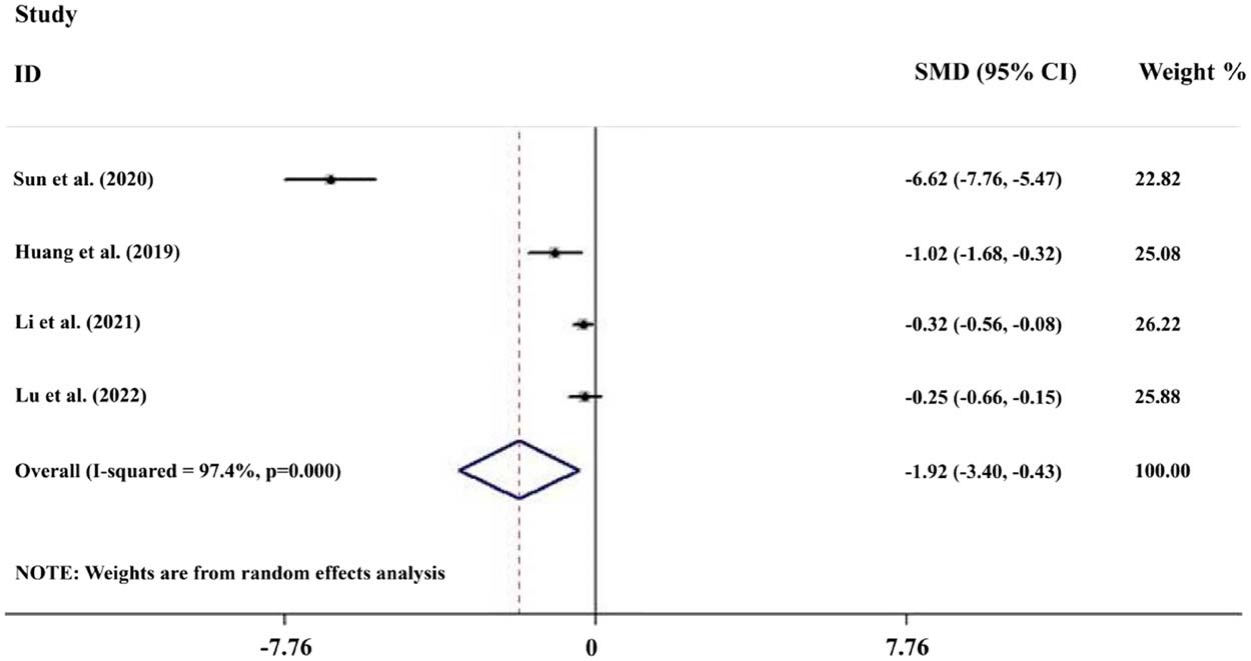
Forest plot of the hospitalization costs.

Analysis of 19 studies with 1609 postoperative inpatients showed that the LOS was significantly lower in the TEAS group (*n*=813) than in the control group (*n*=796) (SMD=−1.00; 95% CI: −1.30, −0.70) (Fig. 2). Further subgroup analyses showed that TEAS reduced LOS for postoperative inpatients in gastrointestinal surgery, lung surgery, gynecological surgery, urological surgery, endoscopic surgery, and surgery in the elderly (Fig. 3A). Analysis of 15 studies with 1404 postoperative inpatients showed that postoperative LOS was significantly lower in the TEAS group (*n*=704) than in the control group (*n*=700) (SMD=−0.70; 95% CI: −0.91, −0.49) (Fig. 4). Further subgroup analyses showed that TEAS reduced the postoperative LOS for postoperative inpatients in gastrointestinal surgery, lung surgery, gynecological surgery, orthopedic surgery, endoscopic surgery, and surgery for the elderly (Fig. 3B). When analyzing four studies with 492 postoperative inpatients, it was found that hospitalization costs were significantly lower in the TEAS group (*n*=246) than in the control group (*n*=246) (SMD=−1.92; 95% CI: −3.40, −0.43) (Fig. 5).

There was evidence of heterogeneity between the studies discussing the effect of TEAS on LOS (*I* ^2^=87.3%, *P*<0.001), postoperative LOS (*I* ^2^=72.5%, *P*<0.001), and hospitalization costs (*I* ^2^=97.4%, *P*<0.001) (Figs 2, 4, 5). To identify the source of the heterogeneity, we conducted a series of subgroup and sensitivity analyses. The leave-one-out analysis did not identify a single article as the source of heterogeneity in the estimated effects of TEAS on LOS and postoperative LOS (SDC, Fig. 1, Supplemental Digital Content 1, http://links.lww.com/JS9/C606, 2, Supplemental Digital Content 1, http://links.lww.com/JS9/C606). In the pool estimates of effect of TEAS on hospitalization costs, exclusion of the studies by Li *et al*. and Lu *et al*., respectively, rendered the results insignificant, indicating these two studies as significant sources of heterogeneity (SDC, Fig. 3, Supplemental Digital Content 1, http://links.lww.com/JS9/C606). However, meta-regression failed to identify any underlying causes of heterogeneity, considering the sample size of the studies. In the subgroup analyses of LOS, we found that heterogeneity was reduced in urological surgery and surgery in the elderly group (Fig. 3). By performing subgroup analyses of postoperative LOS, we found that heterogeneity was reduced in gastrointestinal, gynecological, and orthopedic surgeries (Fig. 3). Thus, the type of surgery was likely to be a source of heterogeneity. Moreover, a series of sensitivity analyses based on the stimulating frequency, acupoint, and quality of study showed that studies carried out TEAS with 2/100 HZ and Neiguanxue were the main sources of heterogeneity of the estimated effect of TEAS on the postoperative LOS (Tables 2 and 3). Additionally, sensitivity analyses carried out by the Trim and Fill method showed no significant differences, indicating the robustness of the results (SDC, Fig. 4, Supplemental Digital Content 1, http://links.lww.com/JS9/C606 and 5, Supplemental Digital Content 1, http://links.lww.com/JS9/C606).

**Table 2:** Sensitivity analysis of the length of stay.

	n	RR	Lci	Uci	I^2^	*P*	Eagger’s test
Stimulation frequency							
100 Hz	18	−0.953	−1.249	−0.657	86.30%	<0.001	0.107
20 Hz	18	−1.480	−1.352	−0.744	86.80%	<0.001	0.208
2 Hz	18	−1.024	−1.336	−0.711	87.90%	<0.001	0.114
2/100 Hz	8	−0.803	−1.158	−0.449	80.70%	<0.001	0.936
2/15 Hz	18	−1.026	−1.340	−0.712	87.80%	<0.001	0.136
2/100 Hz	17	−1.058	−1.386	−0.730	88.20%	<0.001	0.145
Unknown	18	−1.013	−1.327	−0.699	88.20%	<0.001	0.126
Acupoint							
Zusanli	7	−1.268	−1.888	−0.648	91.40%	<0.001	0.318
Hegu	6	−0.705	−1.089	−0.322	79.50%	<0.001	0.877
Neiguan	7	−1.123	−1.500	−0.746	81.60%	<0.001	0.739
Feiyu	16	−0.921	−1.246	−0.596	87.00%	<0.001	0.036
Sanyinjiao	17	−1.058	−1.386	−0.731	88.20%	<0.001	0.145
Grade							
Moderate	7	−0.746	−1.075	−0.418	75.60%	<0.001	0.366
High	12	−1.172	−1.624	−0.719	90.20%	<0.001	0.009

**Table 3:** Sensitivity analysis of the postoperative length of stay.

	*n*	RR	Lci	Uci	*I* ^2^	*P*	Eagger’s test
Stimulation frequency							
2/100 Hz	6	−0.642	−0.793	−0.491	0.00%	0.430	0.589
2/15 HZ	14	−0.711	−0.941	−0.481	74.40%	<0.001	0.255
2/10 HZ	12	−0.724	−0.997	−0.451	76.40%	<0.001	0.174
Acupoint							
Zusanli	6	−0.928	−1.328	−0.528	76.20%	<0.001	0.421
Hegu	7	−0.730	−1.177	−0.284	85.50%	<0.001	0.103
Neiguan	5	−0.730	−0.899	−0.508	0.00%	0.415	0.418
Sanyinjiao	11	−0.706	−0.991	−0.422	78.60%	<0.001	0.264
Shangjuxu	12	−0.693	−0.970	−0.415	77.40%	<0.001	0.290
Grade							
Moderate	4	−0.950	−1.543	−0.356	86.80%	<0.001	0.358
High	11	−0.620	−0.843	−0.396	63.20%	<0.001	0.650

### Risk of bias and study quality assessment

**Figure 6:**
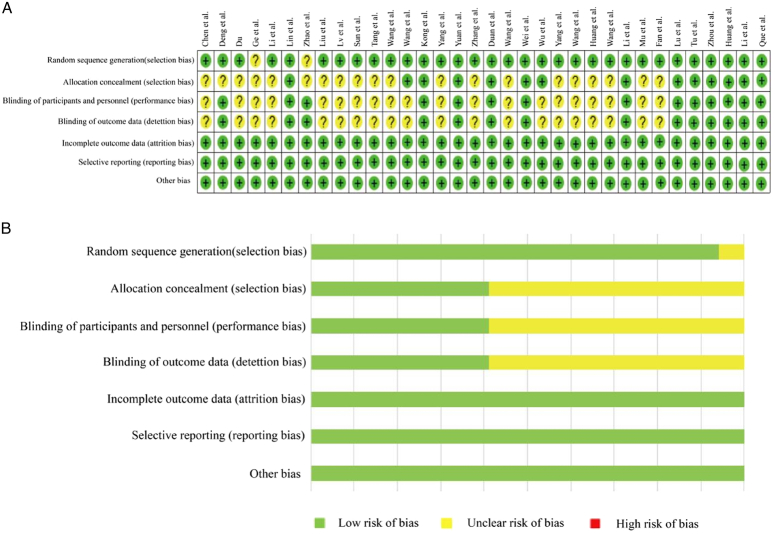
Cochrane risk of bias assessment. Note: A. the risk of bias of each research. B. The risk of bias of all studies. ‘+’ represents low risk; ‘?’ represents unclear risk; ‘-’ represents high risk.

We assessed the RoB of the included studies and rated their study quality using the GRADE approach. The details of the RoB assessment and individual study assessment are shown in Figure 6. As for study quality assessment, 11 (32.4%) studies were rated as high and 23 (67.6%) were rated as moderate (Table 1). The Funnel plot and Egger’s test suggested that there was no evidence of publication bias in studies discussing the effects of TEAS on LOS, postoperative LOS, and hospital costs (SDC, Figs 6–8, Supplemental Digital Content 1, http://links.lww.com/JS9/C606, Supplemental Digital Content 4, http://links.lww.com/JS9/C609).

## Discussion

TEAS combines the traditional Chinese acupuncture theory with modern electrical stimulation technology and is widely utilized in clinics because of its simplicity, stability, and safety^44^. However, previous research has predominantly focused on the clinical effectiveness of TEAS overlooking the significance of hospitalization costs and LOS as crucial factors for both patients and healthcare professionals^2,10,45^. LOS plays a significant role in determining healthcare expenses, and implementing efficient and secure discharge strategies can contribute to reducing financial burden^46^. Moreover, hospitalization costs directly impact inpatients, physician decision-making processes, and the overall economic strain on the nation^46^. Thus, reducing LOS and minimizing hospitalization costs are essential for establishing an effective and economically efficient healthcare system^47^.

Accumulate evidence has emerged over the past 10 years that TEAS plays many beneficial roles in perioperative patients. Firstly, TEAS can improve the gastrointestinal function of postoperative patients, including accelerating the time to the first postoperative gas and bowel movement, the time to the recovery of bowel sounds, the time to the recovery of a normal diet, and reducing the incidence of postoperative nausea and vomiting and paralytic bowel obstruction^48–50^. Mechanistically, acupoint stimulation by TEAS can influence the brain-gut axis, which is a bridge connecting the central nervous system and the gastrointestinal tract. This influence includes the change of brain-gut peptide secretion and the stimulation of the vague nerve and posterior horn of the spinal cord, leading to constructive effects on gastrointestinal sensation, peristalsis and microbiota^11,51,52^. Secondly, TEAS has been reported to reduce the incidence of psychiatric system-related symptoms or disorders, such as anxiety, insomnia, postoperative delirium, postoperative cognitive dysfunction, etc. in postoperative patients^4,53–57^. These roles are closely related to the modulation of endogenous substances by TEAS in perioperative patients. For example, TEAS improves sleep quality by modulating levels of serotonin (5-HT), norepinephrine, cortisol, melatonin, etc.^57^. In addition, TEAS decrease IL-6 and high-sensitivity C-reactive protein levels and increases serum Calcitonin Gene-Related Peptide levels to reduce the cumulative duration of cognitive decline in postoperative patients^58^. Thirdly, TEAS also modulates neurological-related functions, leading to pain relief, less anesthetic drug use, and sedation^59,60^. This may be related to the fact that TEAS induces the release of endogenous opioid peptides (e.g. enkephalins, endorphins, and dynorphins) at the central level to produce analgesia^60^. β-endorphin is the main analgesic substance released by the pituitary gland and plays an important role in self analgesia^61^. Perioperative use of TEAS has been reported to increase serum β-endorphin concentrations in patients^62,63^. Collectively, usage of TEAS could modulate various endogenous substances and regulate nerve system, thus reducing LOS and hospitalization costs in perioperative patients by diverse biological mechanisms. In reviewing the literature, we found that TEAS also plays beneficial roles in the population from countries such as Poland, America, and Turkey, when they are in the perioperative period^64–70^. These studies demonstrated that TEAS relieves pain levels, reduces the use of paroxysmal medications, maintains blood pressure, and reduces the incidence of nausea, indicating that people from other nations besides the Chinese people will also benefit from TEAS in perioperative period ^64–70^. They were not included in our review because they did not evaluate two important indicators of interest, LOS, and cost of hospitalization. However, due to its diverse beneficial effects in the perioperative period in these population, it is reasonable to speculate that TEAS might reduce LOS and hospitalization costs in these patients. Furthermore, as a highest level of evidence, our systematic review and meta-analysis will serve as an important reminder for more attention to the role of TEAS on LOS and the hospitalization costs, to the future studies related to TEAS in the perioperative period worldwide.

Although there was heterogeneity in our results, we conducted subgroup and sensitivity analyses to identify the potential sources of heterogeneity. These included the type of surgery and frequency of stimulation versus stimulation points. We observed that most of the TEAS stimulation intensities used clinically were at the maximum intensity tolerated by patients. However, there were variations in the stimulation frequency and choice of acupuncture points among studies with the same type of surgery. Previous studies have explored different stimulation frequencies in the perioperative period; however, our research has revealed that this approach is inadequate. Therefore, it is essential to identify the specific frequencies and acupuncture points that have the greatest impact on TEAS. Notably, the pooled estimates revealed a pronounced effect of TEAS on hospitalization costs in patients undergoing cesarean section, while TEAS induced decrease in LOS and postoperative LOS were rather prominent in elderly patients and those underwent lung surgery. Furthermore, both studies identified as primary sources of heterogeneity through leave-one-out analysis focused on patients undergoing gastrointestinal surgery. These findings implies that the effect of TEAS may be more prominent in patients undergoing surgeries with greater trauma or those with a poorer baseline condition, although further validation is warranted. Furthermore, we found only one study that included both LOS and postoperative LOS^23^. The inclusion of both metrics not only increases the credibility of the evidence but also allows for a more comprehensive analysis. The current literature has significant limitations in this area, and we strongly encourage future researchers to conduct studies that encompass both LOS and postoperative LOS metrics.

This study has certain limitations. First, the majority of the included trials did not adequately describe allocation concealment, blinding of participants and personnel, or blinding of outcome data. This lack of information raises concerns about the reliability of the results. In addition, some results were drawn from a limited number of studies, leading to a low level of evidence. This restricts the generalizability of the findings and highlights the need for further research. Furthermore, it is worth noting that most of the included studies had intermediate research quality, which may have influenced the reliability of the results. These limitations should be considered when interpreting the findings of this study. Further well-designed trials with a focus on addressing these limitations are necessary to provide more robust and reliable evidence.

## Conclusion

This systematic review and meta-analysis suggests that the use of TEAS during the perioperative period may reduce hospitalization costs, LOS, and postoperative LOS. However, caution is needed when interpreting the results of this review because of the identified bias and the diversity of the findings. Despite the use of TEAS in various surgeries during the perioperative period, current research remains inadequate in comparison to the wide range of surgical conditions. Subsequently, further high-quality studies pertaining to TEAS are required.

## Ethical approval

No patients were involved in this study.

## Consent

No patients were involved in this study.

## Source of funding

None.

## Author contribution

Y.L.: concept development, article writing, and data analysis; J.F. and X.Z.: data collection and data analysis; W.X.: concept presentation and article revision; Z.S. and J.C.: data collection; P.W.: concept presentation and article revision.

## Conflicts of interest disclosure

The authors declare that they have no financial conflict of interest with regard to the content of this report.

## Research registration unique identifying number (UIN)

As required, we registered our systematic review and meta-analysis in the PROSPERO database (registration number: CRD42023416677).

## Guarantor

Peiqin Wang.

## Data availability statement

The data are publicly available.

## Provenance and peer review

Not commissioned, externally peer-reviewed.

## Supplementary Material
