# Supplementary figures and images for: Transcutaneous electrical acupoint stimulation reduces postoperative patients’ length of stay and hospitalization costs: a systematic review and meta-analysis
Source: Int J Surg. 2024 May 22;110(8):5124–35. doi: 10.1097/JS9.0000000000001598 (PMC11325889; doi:10.1097/JS9.0000000000001598)

PRISMA Flowchart


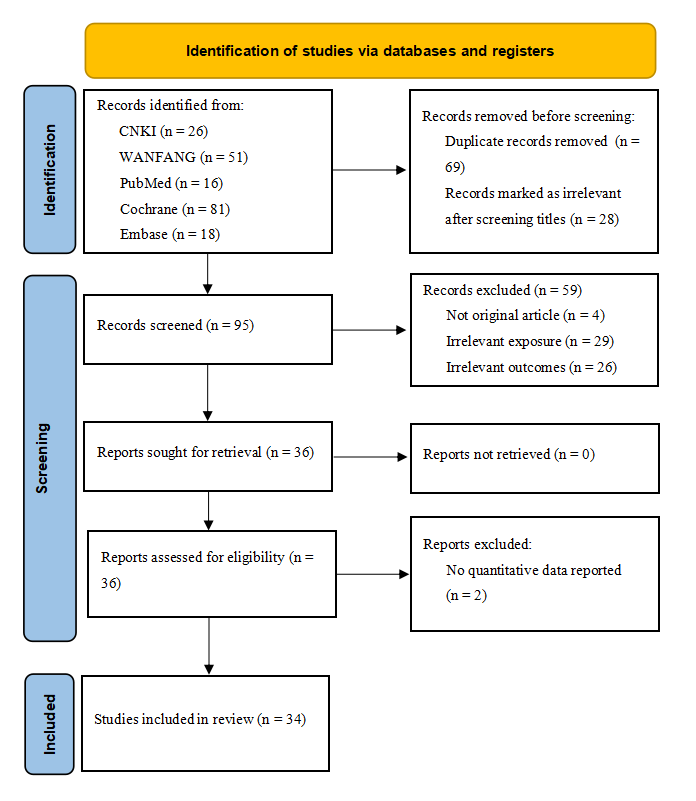

Supplement: Supplementary file 2 [file js9-110-5124-s002.docx]

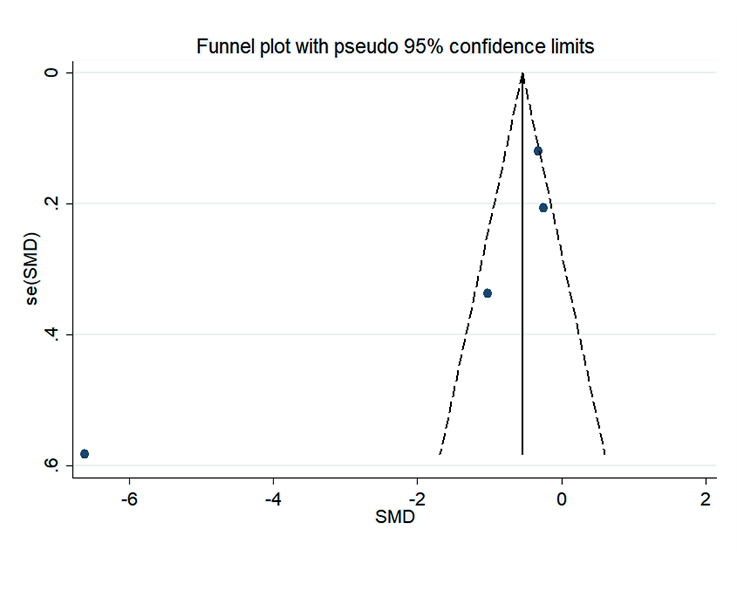


**Figure S8** Funnel plot of the hospitalization costs.

Supplement: Supplementary file 4 [file js9-110-5124-s004.docx]
